# Folk medicine in the northern coast of Colombia: an overview

**DOI:** 10.1186/1746-4269-7-27

**Published:** 2011-09-22

**Authors:** Harold Gómez-Estrada, Fredyc Díaz-Castillo, Luís Franco-Ospina, Jairo Mercado-Camargo, Jaime Guzmán-Ledezma, José Domingo Medina, Ricardo Gaitán-Ibarra

**Affiliations:** 1 Grupo de Investigación en Química de Medicamentos, Facultad de Ciencias Farmacéuticas, Departamento de Farmacia. Universidad de Cartagena. Cartagena de Indias Colombia

**Keywords:** Ethnopharmacological survey, Traditional knowledge, Bolívar-Colombia, Medicinal plants

## Abstract

**Background:**

Traditional remedies are an integral part of Colombian culture. Here we present the results of a three-year study of ethnopharmacology and folk-medicine use among the population of the Atlantic Coast of Colombia, specifically in department of Bolívar. We collected information related to different herbal medicinal uses of the local flora in the treatment of the most common human diseases and health disorders in the area, and determined the relative importance of the species surveyed.

**Methods:**

Data on the use of medicinal plants were collected using structured interviews and through observations and conversations with local communities. A total of 1225 participants were interviewed.

**Results:**

Approximately 30 uses were reported for plants in traditional medicine. The plant species with the highest fidelity level (Fl) were *Crescentia cujete *L. (flu), *Eucalyptus globulus *Labill. (flu and cough), *Euphorbia tithymaloides *L. (inflammation), *Gliricidia_sepium*_(Jacq.) Kunth (pruritic ailments), *Heliotropium indicum *L. (intestinal parasites) *Malachra alceifolia *Jacq. (inflammation), *Matricaria chamomilla *L. (colic) *Mentha sativa *L. (nervousness), *Momordica charantia *L. (intestinal parasites), *Origanum vulgare *L. (earache), *Plantago major *L. (inflammation) and *Terminalia catappa *L. (inflammation). The most frequent ailments reported were skin affections, inflammation of the respiratory tract, and gastro-intestinal disorders. The majority of the remedies were prepared from freshly collected plant material from the wild and from a single species only. The preparation of remedies included boiling infusions, extraction of fresh or dry whole plants, leaves, flowers, roots, fruits, and seeds. The parts of the plants most frequently used were the leaves. In this study were identified 39 plant species, which belong to 26 families. There was a high degree of consensus from informants on the medical indications of the different species.

**Conclusions:**

This study presents new research efforts and perspectives on the search for new drugs based on local uses of medicinal plants. It also sheds light on the dependence of rural communities in Colombia on medicinal plants.

## Background

About 80% of the populations of developing countries continue using traditional resources in health care [1-6]. The main goal of ethnopharmacology is to identify novel compounds derived from plants and animals for use in indigenous medical systems. This knowledge can be used in the development of new pharmaceuticals. Most of the literature in ethnopharmacology describes medicinal plants used by people who have lived in the same ecological region for many generations. Ethnopharmacologists seek ways to improve the ethnomedical systems of the people whom they study by testing indigenous medicines for efficacy and toxicity. Through this kind of work, ethnopharmacology has contributed to the discovery of many important plant-derived drugs [7].

Colombia accounts for approximately 10% of the world's biodiversity and is home to about 50,000 species of plants [8-10], of which only 119 are included in the Colombian Vademecum of Medicinal Plants [11]. The diverse topography of the Colombian territory and the country's wide range of climates have favored the formation of varied habitats. Despite the country's natural richness, the status of scientific knowledge on Colombian flora is still incipient in many aspects. The Andean region represents the largest area in the country. It contains a wide variety of biomes, among which are wetlands. The Pacific region has one of the most important pockets of biodiversity in the world, and the tallest costal mountain in the world (18,700 ft above sea level) is in Colombia's Caribbean region. This mountain, the Sierra Nevada de Santa Marta, has a complex variety of flora and fauna since it is completely separated from other ranges mountain, and it contains many different kinds of habitats [10,12]. It is estimated that only about 70% of the Colombian flora is known, and, on the other hand, there is a worrying number of endangered species. There are also threats due to high levels of water and air pollution and the resulting effects of these on human quality of life and ecosystem health [12-14]. A similar situation has existed regarding traditional knowledge associated with floristic resources in many regions of the country. At different times in Colombia's history, especially between the Conquest and Colonial periods, when people of different cultures have generated an amalgam of varied customs and beliefs which have in turn contributed to create a diverse and valuable cultural heritage that includes the use of medicinal plants. Many species of plants are still used widely to treat common illnesses in practically the entire Colombian territory [15,16]. Despite the great importance that floristic resources represent for the population, the country's health authorities have given the resource little attention, and governmental support for research and development of economically viable alternatives in this field has been scarce. Only in recent years, Colombian authorities have begun to focus on the value of the country's biodiversity and to create mechanisms for its use. Laws have been formulated to regulate the commercialization, production, storage, distribution, and use of medicinal plants. In 1994, for example, a listing of medicinal plants and their approved uses was produced by the *Comisión Revisora de Productos Farmacéuticos-Colombia *(Review Commission for Pharmaceutical Products-Colombia) [11].

The communities that were studied at the north of the department of Bolívar are low-income populations and dedicated mainly to agriculture and ranching. Plantations include grains, fruits, and vegetables, which are the main source of income and employment for men. Large cattle ranches occupy areas that once supported habitats of diverse native plant species that have disappeared and whose number cannot be determined. Handcrafts (hats, mats, musical instruments, tools for agriculture, and others) are made for own consumption or for sale by order. They constitute part of the traditional know-how that is passed from generation to generation. Other sources of income are fishing and forestry, and salt production.

We highlight the community of San Basilio de Palenque investigated in our ethnopharmacological study. San Basilio de Palenque is located in the department of Bolívar, 50 km south of the city of Cartagena de Indias. The community was founded by black slaves who fled and established *palenques *(villages of fleeing slaves, or *cimarrones*) in the northern coast of Colombia, beginning in the fifteenth century. Much later, the term palenque became synonymous with freedom. From the many *palenques *that existed in the Colonial period, San Basilio is the only one that has survived, and it has struggled to maintain its cultural identity. San Basilio de Palenque is the cradle and testimony of the rich African traditions in Colombia.

San Basilio, known also as Palenque, is famous for its symbol, the palenqueras, dark-skinned women dressed in multicolor dresses who carry fresh fruit and traditional candies in large bowls (palanganas) on their heads and sell them in cities such as Cartagena.

San Basilio de Palenque conserves its ethnic attributes and its spiritual outlook of life and death, for example its musical rituals expressed in the celebration of saints, its complex funeral rituals known as *lumbalú*, and the use of traditional medicines including medicinal plants. For all of the reasons above, San Basilio de Palenque is a strong cultural influence throughout the Colombian Caribbean, and it symbolizes the struggle of Afro-Colombian communities to achieve freedom, ethnic equality, peaceful coexistence, and the recognition of their cultural diversity.

The Colombian State, in collaboration with these communities, has formulated and developed plans to preserve, conserve, and protect the different popular expressions and knowledge that constitute the communities' identity. However, factors such as racial discrimination, forced migrations resulting from the violence that has affected Colombia for more than 50 years, acculturation, and the lack of plans for cultural transmission, have affected the expression of traditions that are unique to the regions of northern Colombia, including the passing of knowledge related to traditional medicines, in detriment to the cultural stability of communities and the cultural diversity of the region [12].

The Colombian Caribbean region has a unique combination of geographical and topographical attributes that has given it an extraordinary diversity of flora and fauna. Moreover, considering the cultural diversity of its people, this region has a great pharmacological reserve based on its natural resources. Some characteristic species of this part of Colombia are known commonly as pringamosa (*Urera baccifera *(L.) Gaudich.), trupillo (*Prosopis juliflora *(Sw.) DC.), caracolí (*Anacardium excelsum *(Kunth) Skeels), clemón (*Thespesia populnea *(L.) Correa), dividivi (*Libidibia coriaria *(Jacq.) Schltdl), matarratón (*Gliricidia*_*sepium*_(Jacq.) Kunth), totumo (*Crescentia cujete *L.), ají (*Capsicum annuum *L), ajo (*Allium sativum *L.), anamú (*Petiveria alliacea *L.), guayaba (*Psidium guajava *L.), mamey (*Mammea americana *L.), tomate (*Lycopersicon esculentum *Mill.) and orégano (*Lippia graveolens *Kunth.) [9,10,15,17].

**Figure 1:**
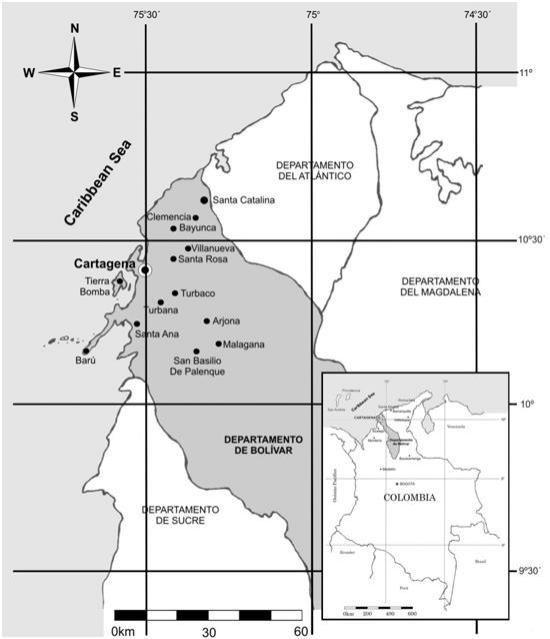
**Map of the surveyed area and collection sites**.

The common interest of many Colombian researchers of preserving this cultural tradition has led to interdisciplinary work among several institutions, for example with the purpose of finding natural-therapy alternatives to several illnesses [10,18,19]. For instance, research groups in several disciplines have worked with small communities in the department of Bolívar on the recovery of botanical knowledge in small towns. The location of the main areas can be seen in Figure 1.

The use of medicinal plants as an alternative medicine for the Colombian population has been approved by the government recently, and the Social Protection Department has produced the Colombian Vademecum of Medicinal Plants, which lists the species that have been approved for a specific use and their verified pharmacological activity, main components, instructions for use, toxicity, counter indications, and available pharmaceutical forms [11].

The work presented in this paper can be included into the general context of basic and applied ethnopharmacology because it was conceived to document the traditional knowledge on botanical medicine from Colombia's Caribbean region. Although other valuable investigations have been carried out on indigenous communities, black communities and rural communities in other regions of Colombia with the support of universities and non-government organizations [13], in the Atlantic Coast, and more concretely in the department of Bolivar, the development of ethnobotanical investigations is scant. Considering the enormous floristic wealth of this region and the cultural diversity of its communities, it is crucial keep documented this cultural heritage to avoid dissapearing this valuable knowledge forever. Deforestation and unsustainable farming practices represent additional threats for the medicinal flora and the traditional practices that depend on it.

## Methods

### Description of the study area

The geographical region under study includes several small towns of the department of Bolívar in the northern coast of Colombia (Figure 1). The study region is located between 75°15' and 75°45' West longitude and between 10°10' and 10°40' North latitude, forming a quadrant that includes Turbaco, Ballestas, Malagana, San Basilio de Palenque, Santa Rosa, Bayunca, Barú and the insular region of Tierrabomba to the southwest of Cartagena. The mean annual temperature oscillates between 25°C and 32°C, and the relative humidity is about 80%. The rainy season occurs during the months of January and February, and rain persists until May. During June, July, and August is the "San Juan's veranillo", a dry season with isolated rains. A rainy season occurs also from September to November. December presents fresh winds and few rains [20].

Natural forests in the region have almost completely disappeared due to economic activities. This has changed the local use of the flora and has caused the disappearance of plant species, especially in the proximity of mountains. In areas near Turbaco and Arjona, besides agriculture and cattle ranching, non-sustainable exploitation of quarries also exist which generate large quantities of residues that are discharged into water sources [9].

### Survey participants and interviewing

We conducted ethnopharmacological semi-structured interviews between 2007 and 2010. After obtaining prior informed consent, participants were interviewed according to TRAMIL participative ethnopharmacological interviews, with some modifications [21]. In total, 1225 participants (884 women, 341 men), all older than 40, were interviewed. The questionnaire included both quantitative and qualitative elements. Questions were designed in order to collect information about principal illnesses, medicinal-plant knowledge, and different uses of medicinal plants. More specifically, the standardized format of the ethnopharmacological interview included questions related to the type of disorders treated, a detailed explanation about the manner of treatment, the plants used when the health problem occurred, as well as common names, the parts of the plant used, medicine preparation, plant condition (fresh or dried), dosage, and routes of administration [4,7,20]. On some occasions, we visited the areas of plant recollection with the respondents, which allowed us to get a direct knowledge of some species.

### Plant collection and identification

Whenever possible, common names and specimens were taken from the survey respondent(s) for verification as the correct species and identification of plant material. Vouchers were collected and numbered during the interviews. Specimen identification was carried out by comparison with authentic samples. The vouchers were deposited at the Institute of Botany of the *Universidad de Antioquia-Colombia *(HUA) and Botanical Garden in Cartagena (JBC).

The collected data were analysed using statistical tools that allowed us to relate the use of the species with a particular popular knowledge about them. We considered the predominance of values according to a central tendency. The fidelity level (Fl) of plant uses mentioned by the interviewed groups [22-24] was calculated according to the following formula:

Fl=(Ip∕Iu)×100

Where *I_p _*is the number of informants that used this part of a plant for a particular use; *I_u _*is the number of informants that used plants as medicine for this use. The formula was applied in order to compare data from different uses of plants where the survey was performed.

The Informant Agreement Ratio (IAR) was calculated according to the following formula:

IAR=(Ur-Npu)∕(Ur-1)

*Ur *is the reported uses and *Npu *is the number of plants uses. Here, consensus is measured with reference to increased frequency of occurrence of the category of ailments. These values were a powerful tool that, together with searches in available bibliographical databases, facilitated the further development and depuration of the information [22-24]. When this value is equal to one, all respondents agree on a single species for a particular use or health problem.

## Results and discussion

In our study, a total of 1225 heads of family were interviewed in 8 small towns of the northern department of Bolívar. Many of the interviews were accompanied by supplemental information with specific details of the species studied, such as location, means of collection, disease symptoms, botanical identification, and personal testimonies of the people related to their living experiences.

Men seemed to have less knowledge than women about traditional medicine, probably because they spend less time than women on tasks related to family health. The average age of the participants was 54. Approximately 39 plants were reported by informants as the most used as notable healing remedies, with 33 medicinal uses. In order to analyze the data, all ailments were categorized into 14 major groups based on the system or organ of the human body affected (Table 1). In Table 2 lists the main species used, their botanical and family name (in alphabetical order by scientific name), collection number, common name, popular usage, part plant used, and a simple description of the manner of preparation and administration.

**Table 1:** Groups of reported illnesses.

**No**.	Group	Illness
1	Cardio-vascular diseases	Hypertension, hematoma, edema.
2	Central nervous system complaints	Sedative, headaches, nervousness.
3	Ear ailments	Paint in ear (earache).
4	Gastro-intestinal system disorders	Stomach ache, abdominal pain, colic, flatulence, gastric bloating, vomiting, indigestion, diarrhea, constipation, helminth infection, intestinal parasites.
5	Hepatic diseases	Hepatic pain, hepatitis, blood purifier.
6	Infections	Fever, abscess.
7	Inflammation	Inflammation, arthritis.
8	Metabolic diseases	Diabetes, anemia.
9	Mouth and dental disorders	Toothache, infection in mouth.
10	Ophthalmologic complaints	Pain in eyes, conjunctivitis, eye injuries.
11	Respiratory tract diseases	Cough, bronchitis, flu, cold, asthma, phlegm (cold and wet), cogged nose.
12	Skin affections	Eczema, pruritic ailments, abscess or other inflamed wounds, boils, dermatosis presumably caused by fungal or yeast infections, dry skin condition
13	Urinary ailments	Kidney stones, kidney pain, urethritis, dysuria, urinary ailments.
14	Other syndromes	Insomnia, body ache, malaria, weakness, injury, sprain, burn, lice, menstrual pain (dysmenorhea), insect and snake bites, cardiovascular diseases, evil eye (*mal de ojo*).

**Table 2:** Summary of ethnopharmacological background and medicinal plants

Scientific name Family [voucher number]	Vernacular name	Principal medicinal indication/(Fidelity Level)^♣ ^	Part used (condition)	Mode of preparation	Way of Administration
*Allium sativum *L.Liliaceae	Ajo	Intestinal parasites (23) ^♣,*,§^	Bulbs (fresh)	Decoction or fresh	Orally
		flu (12)			
		Cardiovascular diseases (4)			
*Aloe vera *(L.) Burm.f.^†^ Liliaceae	Sábila	Flu (24) ^♣,*,§^	Internal part of the leaf (fresh)	Liquefied with honey (syrup) or decoction	Orally
		Phlegm (14)			
		Inflammation by burns (35) ^♣,^*		Fresh pulp	Applied locally
*Alpinia purpurata *K. Schum Zingiberaceae [HUA 140922]	Matandrea	Headache (34)^♣^, *	Leaf (fresh)	Macerated, cataplasm	Applied locally
		Common cold (21) ^♣,^*		Decoction	Bath
*Ambrosia cumanensis *Kunth.^†^ Asteracea [HUA 140954]	Ajenjo	Intestinal parasites (2)	Leaf (fresh)	Decoction	Orally
*Ambrosia peruviana *Willd.Asteraceae [JBC 6090]	Artemisa and altemisa	Headache.^§ ^(5)	Leaf (fresh)	Macerated (poultice)	Applied locally
		Common cold (21) ^♣,^*		Decoction	Bath
*Anacardium occidentale *L.Anacardiaceae	Marañon	Diabetes (17)	Fruit	Juice	Orally
*Annona muricata *L.Annonaceae [JBC 1618]	Guanabana	Fever (25) ^♣,^*	Leaf (dried or fresh)	Decoction	Orally
		Inflammation (22) ^♣,^*	Leaf (fresh)		Bath
*Annona purpurea *Moc. & Sessé ex Dunal Annonaceae [JBC 5121]	Matinbá	Evil eye (25) ^♣,^*			
		Inflammation (4)			
		Body ache (8)	Leaf (dried or fresh)	Decoction	Bath
*Aristolochia anguicida *Jacq.Aristolochiaceae [JBC 12473]	Capitana and contracapitana	Pruritic ailments (22) ^♣,^*	Stem or lianas (dried)	Macerated in rum for about 20 days	Applied locally
		Snake bite (34)^♣^*			
*Bauhinia aculeata *L.Caesalpiniaceae [JBC 002643]	Pata de vaca	Diabetes (12)	Leaf (dried or fresh)	Decoction	Orally
*Chenopodium ambrosioides *L.Chenopodiaceae [HUA 140924]	Yerba santa	Internal parasites (24) ^♣,*,§^	Aerial parts (fresh)	Infusion	Orally
*Crescentia cujete *L.^†^ Bignoniaceae [JBC 47413]	Totumo	Flu (29)^♣^,*	Internal part of the fruit	Decoction	Orally
*Eucalyptus globulus *Labill.^†^ Myrtaceae [JBC 005233]	Eucalipto	Flu (21) ^♣,*,§^	Leaf (dried or fresh)	Decoction	Orally
*Euphorbia tithymaloides *L.Euphorbiaceae [JBC 3325]	Pitamorreal	Earache (16)	Leaf (fresh)	Macerated	Application Locally
		Inflammation (24)^♣,^*		*Soasadas *^‡ ^(poultice)	
		Common cold (7)		Decoction	Bath
*Gliricidia sepium *Steud.Fabaceae [HUA 140962]	Matarratón	Fever (29) ^♣,^*	Leaf (fresh)	Decoction	Bath
		Body ache (10)			
		Pruritic ailments (44) ^♣,^*			
*Guazuma ulmifolia *Lam.Malvaceae [JBC 4539]	Guásimo	Flu (8)*, ^§^	Leaf (dried)	Decoction	Orally
		Inflammation (21) ^♣,^*	Latex	Fresh	Applied locally
		Constipation (12)	Bark (dried)	Decoction	Orally
*Heliotropium indicum *L.Boraginaceae [JBC 3691]	Rabo de alacrán and verbena	Internal parasites (25) ^♣^,*	Leaf (fresh)	Decoction	Orally
		Pruritic ailments (21) ^♣,^*			Applied locally
*Justicia chaetocephala *(Mildbr.) Leonard Acantaceae [JBC 3797]	Chingamochila	Inflammation (6)	Leaf (fresh)	Macerated	Applied locally
		Urinary ailments (25) ^♣,^*		Decoction or infusion	Orally
*Luffa operculata *(L.) Cogn.Cucurbitaceae [JBC 278]	Estropajito pequeño	Sinusitis (30)^♣^*	Fruit (dried)	Decoction	Nose instillation
		Cogged nose (12)			
*Malachra alceifolia *Jacq.Malvaceae [JBC 3324]	Malva	Inflammation (21)^♣^,*	Leaf (fresh)	Decoction	Applied locally
		Fever (12)			Bath
*Mangifera indica *L.Anacardiaceae [HUA 140952]	Mango	Indigestion (25)^♣^,*	Leaf (fresh)	Decoction	Orally
		Inflammation (2)	Bark (dried)		
*Matricaria chamomilla *L. ^†^ Asteraceae	Manzanilla	Colic (44)^♣, *, §^	Aerial parts (fresh)	Infusion or decoction	Orally
		Nervousness (22) ^♣,^*		Infusion	Orally
		Conjunctivitis (22) ^♣,^*		Decoction	Eyes instillation
*Mentha sativa *L.Labiatae	Hierba Buena	Nervousness (55)^♣^,*	Leaf (dried or fresh)	Decoction	Orally
*Momordica charantia *L. M. Nee.Cucurbitaceae [HUA 140953	Balsamina	Intestinal parasites (21) ^♣,^*	Aerial parts (fresh)	Infusion	Orally
		Fever (5)			
		Pruritic ailments (12)	Leaf (fresh)	Macerated	Applied locally
*Murraya exotica *L.Rutaceae [JBC 098]	Azahar de la India	Toothache (35)^♣^*	Leaf (fresh)	Macerated	Applied locally
		Fever (13)	Aerial parts	Decoction	Bath
*Ocimun basilicum *L.^†^ Lamiaceae [JBC 000441]	Albahaca	Dry skin condition (25) ^♣,^*	Leaf (dried or fresh)	Decoction	Bath
		Common cold (21) ^♣,^*			
*Ocimum tenuiflorum *L.Lamiaceae [JBC 000312]	Toronjil	Flatulence (21) ^♣,^*	Aerial parts (fresh).	Decoction or infusion.	Orally.
		Nervousness (13)			
*Origanum mejorana *L.^†^ Lamiaceae	Mejorana	Flatulence (30) ^♣^,*	Aerial parts (fresh)	Macerated and decoction	Orally
*Origanum vulgare *L.^†^ *Labiatae* [JBC 012]	Orégano	Earache (48) ♣,*	Leaf *Soasadas*‡	Instillation	Application Locally
*Petiveria alliacea *L.Phytolaccaceae [HUA 140961]	Anamú	Fever (38) ^♣,*,§^			
		Asthma (21) ^♣,^*	Leaf (dried or fresh)	Decoction Decoction	Orally Inhalation of vapor
*Plantago major *L.^†^ Plantaginaceae [HUA 140926]	Llantén	Kidney pain (31)^♣^	Leaf (fresh)	Decoction infusion	Orally
		Abdominal pain (12)		Macerated/decoction	
		Eye injuries (25) ^♣,^*		Infusion	Eye instillation
*Psidium guajava *L.Myrtaceae [HUA 140931]	Guayaba	Nervousness (18)	Leaf (fresh)	Decoction	Orally
		Diarrhea (34)^♣^, *			
*Ricinus communis *L.Euphorbiaceae [JBC 465]	Higuereta	Common cold (22)^♣^, *	Leaf (fresh)	Decoction	Bath
*Russelia equisetiformis *Schlecht. & Cham.Scrophulariaceae [JBC 000353]	Cola de Caballo	Kidney stones (25) ^♣,^*	Entire plant (fresh).	Decoction	Orally
		Constipation (16)	Aerial parts		
*Sambucus nigra *L.Caprifoliaceae [JBC 5]	Salvia peluda	Boils (45) ^♣,^*	Leaf (fresh)	Macerated	Application Locally
		Flatulence (22) ^♣,^*		Decoction	Orally
*Solanum americanum *Mill, W. D'Arcy Solanaceae [HUA 140962]	Hierba mora	Toothache (38) ^♣,^*	Leaf (fresh)	Macerated and decoction (poultice)	Application Locally
		Skin affections (22) ^♣,^*			Bath
*Tabebuia rosea *DC.Bignoniaceae [JBC 3069]	Apamate	Skin affections (25) ^♣,^* Fever (7)	Bark and stem (dried)	Decoction	Bath Orally
*Terminalia catappa *L., J. Espina.Combretácea [JBC 977]	Almendro(a)	Skin affections (21) ^♣,^*	Leaf (fresh)	*Soasadas*^‡ ^(poultice)	Topic Application
*Tithonia diversifolia *A. Gray.Asteraceae [JBC 000038]	Arnica	Skin affections (10)	Leaf (fresh)	Macerated (poultice)	Application Locally

^♣^Indication with Fl > 20%, uses mentioned by ≥ 20% of the respondents that mentioned this illness

**^† ^**Medicinal Plants Accepted in Colombia as Alternative Pharmacological Medicine [10]

**^‡ ^**Previous to application, leaves can also be heated over fire

**^§ ^**Significant traditional Tramil Uses [21]

Our results suggest that people have done a coherent use of plants for medicinal goals in the study region. Species with the largest number of reported uses were *Allium sativum *L. (illness: intestinal parasites), *Aloe vera *(L.) Burm.f. (flu), *Alpinia purpurata *K. Schum (headache and common cold), *Ambrosia cumanensis *Kunth (common cold), *Annona muricata *L. (fever and inflammation), *Annona purpurea *Moc. & Sessé ex Dunal (evil eye), *Aristolochia anguicida *Jacq. (pruritic ailments and snake bite), *Chenopodium ambrosioides *L. (internal parasites), *Crescentia cujete *L. (flu), *Eucalyptus globulus *Labill. (flu), *Euphorbia tithymaloides *L. (inflammation), *Gliricidia sepium *(Jacq.) Kunth (pruritic ailments and fever), *Guazuma ulmifolia *Lam. (inflammation), *Heliotropium indicum *L. (internal parasites and pruritic ailments), *Justicia chaetocephala *(Mildbr.) Leonard (Urinary ailments), *Luffa operculata *Cogn. (Sinusitis), *Malachra alceifolia *Jacq. (inflammation), *Mangifera indica *L. (indigestion), *Matricaria chamomilla *L. (colic, conjunctivitis and nervousness), *Mentha sativa *L. (nervousness), *Momordica charantia *L. M. Nee. (intestinal parasites), *Murraya exotica *L. (toothache), *Ocimum basilicum *L. (dry skin condition and common cold), *Ocimum tenuiflorum *L. (Flatulence), *Origanum majorana *L. (flatulence), *Origanum vulgare *L. (earache), *Petiveria alliacea *L. (fever), *Plantago major *L., R. Liesner (kidney pain and eye injuries), *Psidium guajava *L. (diarrhea), *Ricinus communis *L. (Common cold), *Russelia equisetiformis *Schlecht. & Cham. (kidney stones), *Solanum americanum *Mill. (toothache), *Tabebuia rosea *DC. (skin affections) and *Terminalia catappa *L. (skin affections).

The plant families mostly used by inhabitants were Asteraceae, Lamiaceae, Anacardiaceae, Annonaceae, Bignoniaceae, Cucurbitaceae, Euphorbiaceae, Liliaceae and Myrtaceae. 82% of the plants that are administered internally are prepared as a decoction, infusionor by extraction of the juice of fresh leaves after mashing the plant in some water. For external uses such as dermatological problems, poultice is the preferred form of application.

The highest IAR values (Informant Agreement Ratio, IAR = 0.96), which indicated the major consensus among informants, were established for skin problems like pruritic ailments, boils, dry skin condition and dermatosis presumably caused by fungal or yeast infections and inflammations. Other IAR values were as follows: respiratory tract diseases and cough (0.95), gastro-intestinal system disorders (0.92), central nervous system complaints (0.89), infections (0.85), ear ailments (0.85), and urinary ailments (0.85) (Table 3). Our results showed a great similarity to the corresponding data on morbidity given by the Office of Health of the department of Bolívar for the years 2006-2008 [25,26].

**Table 3:** Rank-ordered list of folk herbal remedies according to category of ailments for which they were employed.

Category	Uses reports (*Ur*)	Number of plants uses (*Npu*)	Informant Agreement Ratio (*IAR*)
Skin affections	285	11	0,96
Inflammation	165	7	0,96
Respiratory tract diseases	215	12	0,95
Gastro-intestinal system disorders	122	11	0,92
Central nervous system complaints	55	7	0,89
Infections	35	6	0,85
Ear ailments	14	3	0,85
Urinary ailments	14	3	0,85
Other syndromes	85	15	0,83

The parts of the plant most used for medicinal purposes were, in decreasing order were: leaves (63%), the complete aerial parts (15%), bark (8%), fruits (6.5%) and other organs (7.5%). Oral ingestion was found to be predominantly the way of use of the medicinal plants in this study. Decoction and infusion (almost always in water) were the main methods of preparation, either for oral or for external administration. For topical use, the most important methods were a direct application and poultice. In some instances, other ingredients were added to the preparation, such as honey, sugar, salt, oil, and brown-sugar loaf (commonly known as *panela*).

The observations emanating from the present survey need to be substantiated with other pharmacochemical studies in order to scientifically evaluate or validate their popular uses. However, for some species, there is evidence in the literature that the mode of application being practiced by the local people is likely to be effective. For example, our group reported several furanonaphthoquinones isolated from the bark and stem of *Tabebuia sp*., and these compounds have been related to antimalarial activity [27-29]. In 2002, two diterpenoids and aristolochic acid I were isolated from the methanolic extract of the stem of *Aristolochia anguicida *Jack, and were also associated to some treatments [30]. Other uses have been validated and reported in *Farmacopea Vegetal Caribeña *for the Tramil group [21,31] and other important research groups in Colombia [32-35].

## Conclusions

There is a great variety of medicinal plants in the department of Bolívar (Colombia), and they have been used traditionally by the population for the treatment of their illnesses. This knowledge has been passed from generation to generation. The multiple uses reported in this study indicate that scientific investigations are useful in the validation of traditional medicinal practices in this region, which can allow obtaining and developing new therapeutic agents from Colombian plants.

This study showed a high level of coincidence among respondents regarding the use of medicinal plants in the region. The plants used for the most frequent illnesses are *Aristolochia anguicida *Jacq., *Gliricidia sepium *(Jacq.) Kunth, *Ocimum basilicum *L. *Tabebuia rosea *DC. and *Terminalia catappa *L. for skin affections and *Annona muricata *L., *Euphorbia tithymaloides *L., *Guazuma ulmifolia *Lam. and *Malachra alceifolia *Jacq. for inflammation.

Quantitative studies on traditional uses of plants as medicines, such as the one we presented here, are particularly important because they address crucial aspects of a region's biological and cultural diversity.

## Competing interests

The authors declare that they have no competing interests.

## Authors' contributions

JGL and HGE were responsible for the field work and for the first draft of the manuscript. HGE participated on the coordination and guidance of the research. All authors have written, read and approved the final version of the manuscript.
